# Opportunistic depredation of songbird nestlings by female praying mantids (Mantodea: Mantidae)

**DOI:** 10.1002/ece3.9643

**Published:** 2022-12-13

**Authors:** Mahmood Kolnegari, Antonio Fasano, Khalil Zareie, Connor T. Panter

**Affiliations:** ^1^ Avaye Dornaye Khakestari Institute Arak Iran; ^2^ Università degli studi di Salerno Fisciano Italy; ^3^ Freelance researcher Manujan County Iran; ^4^ School of Geography University of Nottingham Nottingham UK

**Keywords:** chicks, mantidae, opportunistic behavior, passeriformes, predation, trophic interactions

## Abstract

Praying mantids (class Insecta, order Mantodea) are a group of predatory insects comprising approximately 2500 described species, that occur across all continents except Antarctica, with the greatest species diversity in tropical and subtropical regions. Mantids predominantly prey on other invertebrates but are known to feed on small vertebrates. During April and May 2021, we observed mantid feeding events in Manujan County, Kerman Province in southern Iran. Two distinct feeding events were observed where female European Mantids (*Mantis religiosa*) preyed on Purple Sunbird (*Cinnyris asiaticus*) and Crested Lark (*Galerida cristata*) nestlings. In addition, we collated information from online searches of mantids feeding on nestlings elsewhere in the world, revealing two more observations. In Taiwan, a Giant Asian Mantid (*Hierodula patellifera*) was recorded preying on a nestling Warbling White‐eye (*Zosterops japonicus*) and in Brazil, a mantid (*Stagmatoptera* sp.) was recorded feeding on a nestling White‐throated Seedeater (*Sporophila albogularis*). To date, the only existing scientific evidence of praying mantids feeding on passerine nestlings was recorded in 1922. We propose two potential explanations for the observed trophic interactions between mantids and passerine nestlings: (1) during egg production female mantids, especially those in poor physical condition, may opportunistically feed on nestlings in order to increase fecundity via nutrient gain and (2) mantids may initially be attracted toward the nest by parasitic or coprophagous insects, as a result of poor nest sanitation, and subsequently prey on nestlings after detecting movements. Our unusual observations represent the first records of praying mantids feeding on nestling passerines in nearly 100 years.

## Graphical abstract

We describe the unusual and previously undescribed observations of female European Mantids depredating Purple Sunbird and Crested Lark nestlings in southern Iran.

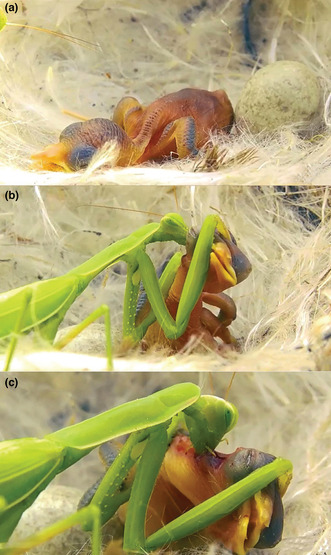

## 1 INTRODUCTION

Praying mantids (class Insecta, order Mantodea) are a group of predatory insects comprising approximately 2500 described species (Anderson, 2022; Otte et al., 2022) and are present in all continents, except Antarctica, with the greatest species diversity in tropical and subtropical regions (Foottit & Adler, 2018; Prete et al., 1999). Between species, praying mantids display a myriad of hunting tactics spanning from active hunting strategies to ambushing prey (Pickard et al., 2021). Praying mantids catch their prey using a pair of front legs modified into raptorial limbs equipped with spines (Loxton & Nicholls, 1979). Several mantid species have developed unique strategies which have allowed them to adapt to a specific ecological niche, e.g., impaling prey (Rivera & Callohuari, 2020), others are highly adaptable generalist predators (Bosse et al., 2022; Fukudome & Yamawaki, 2016). Compared with most other arthropods, mantids are large in size allowing for the capture of small vertebrates including amphibians, rodents, reptiles, fishes and birds (Battiston et al., 2018; Costa‐Pereira et al., 2010; Hernandez‐Baltazar et al., 2020; Jehle et al., 1996; Valdez, 2020).

One of the first observations of mantids hunting vertebrates dates back to the 13th century when Albert Magnus, a German scientist and philosopher, documented a praying mantid hunting a snake (Prete et al., 1999). For other potential prey groups, such as birds, scientific evidence tends to be anecdotal and mostly from members of the American general public (see Nyffeler et al., 2017). A study by Nyffeler et al. (2017) reviewed 147 incidents of praying mantids capturing small birds. The recorded incidents were related to 12 mantid species (genera: *Coptopteryx* Saussure, 1869; *Hierodula* Burmeister, 1838; *Mantis* Linnaeus, 1758; *Miomantis* Saussure, 1870; *Polyspilota* Burmeister, 1838; *Sphodromantis* Stål, 1871; *Stagmatoptera* Burmeister, 1838; *Stagmomantis* Saussure, 1869 and *Tenodera* Burmeister, 1838) and 24 bird species mainly from the family Trochilidae (including Hummingbirds). Other than a single observation by Morse (1922), who encountered a mantid preying on a “tiny naked” Yellow‐rumped Thornbill (*Acanthiza chrydorrhoa*), there has been little evidence of mantids actively preying on nestlings within the nest. Here, we build on the evidence first proposed by Morse (1922) and report mantids feeding on nestling birds in southern Iran, along with similar observations by non‐experts on social media and news outlets.

## 2 FIELD OBSERVATIONS

Field observations were conducted on 2 April and 17 May 2021 in the Manujan County (27°30′N, 57°52′E; 337 m above sea level), Kerman Province, southern Iran. We used a Canon 5D Mark п professional camera to record predation events. Mantids were identified to species‐level using Giglio‐Tos (1927) and Battiston et al. (2010). Nestling birds were identified from the following: Svensson (2011); Dickinson & Christidis (2014); Kolnegari & Hazrati (2018).

In addition to our field observations, we conducted online searches on Google (www.google.com) to collate information on mantids predating nestling birds documented by members of the general public. We searched using the following key search terms: “predation”, “hunting”, “chicks”, “nest”, “bird”, “mantid” and “mantis”. Beside English, online searches were performed in Arabic, Chinese, French, Italian, Persian and Spanish.

**FIGURE 1:**
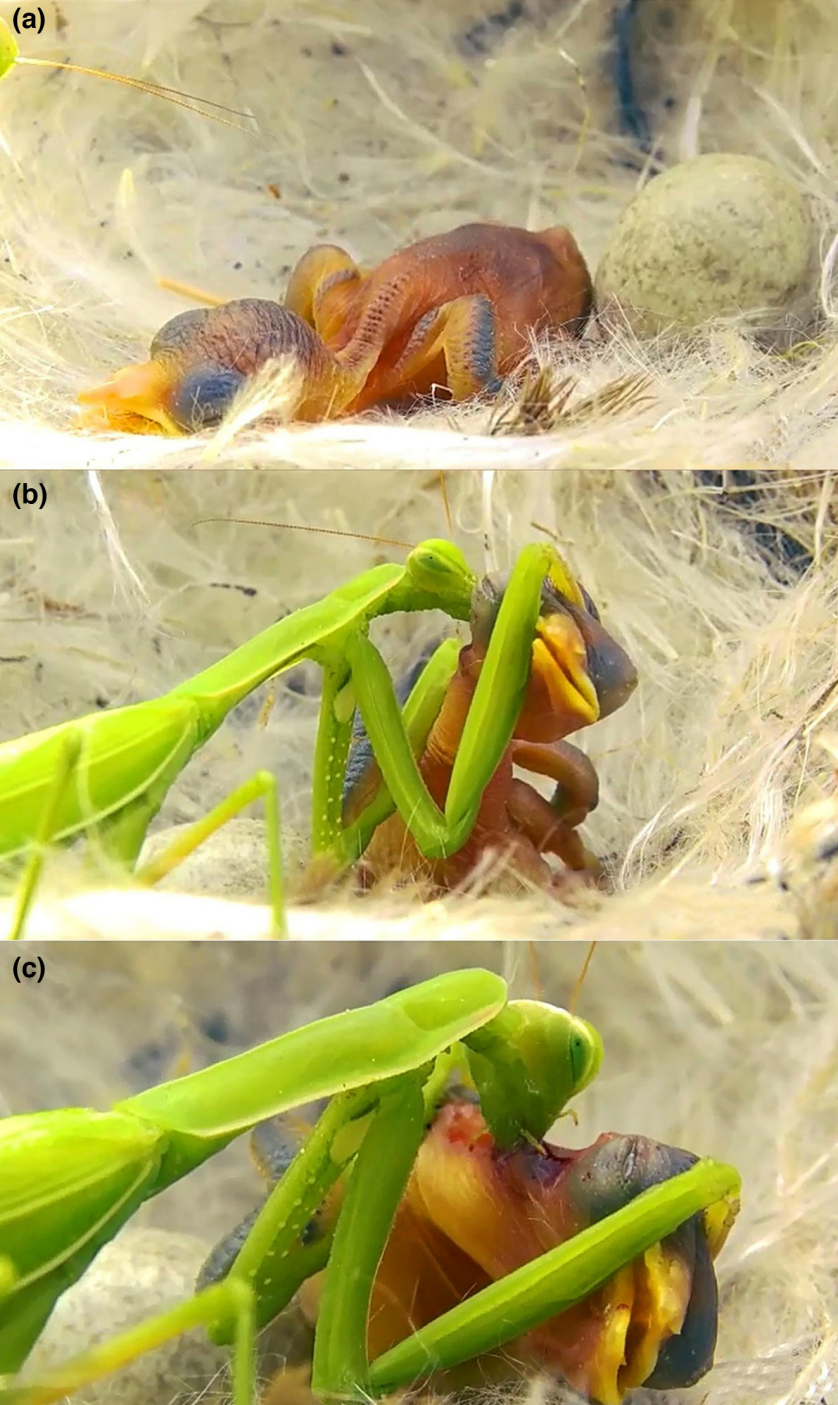
Female European Mantid (*Mantis religiosa*) predating a Purple Sunbird (*Cinnyris asiaticus*) nestling in Manujan County (27°30′N, 57°52′E), Kerman Province, southern Iran. (a) Purple Sunbird nestling recently hatched from the egg, (b) female European Mantid grasps nestling and (c) begins feeding.

From our field observations, we documented two events where female European Mantids (*Mantis religiosa*) were observed feeding on nestling birds. The female mantids fed on Purple Sunbird (*Cinnyris asiaticus*) and Crested Lark (*Galerida cristata*) nestlings that were approximately a few days old (Table 1; Figure 1). The Purple Sunbird nest was located on a Christ's Thorn Jube tree (*Ziziphus spina‐christi*), whereas the Crested Lark nest was located at ground‐level. On both occasions, the female mantids appeared to prey on nestlings opportunistically. The mantids were located close to the nest locations and hunting was initiated by the movements of the nestlings within the nest. We suggest that the mantids were behaving opportunistically as prior to this, the mantids appeared unaware of the nestlings' presence. Once the mantids seized the chicks, they began feeding and dragged the chicks out of the nests.

**TABLE 1:** An overview of praying mantid predation events (*N* = 4) on passerine nestlings in Iran, Taiwan and Brazil between 2018 and 2021.

No.	Mantid species	Bird species	Location	Date	Reference
1	European Mantid (*Mantis religiosa*)	Purple Sunbird (*Cinnyris asiaticus*)	Iran	2021	Filmed by Khalil Zareie (see Figure 1)
2	European Mantid	Crested Lark (*Galerida cristata*)	Iran	2021	Direct observation by Khalil Zareie
3	Giant Asian Mantid (*Hierodula patellifera*)	Warbling White‐eye (*Zosterops japonicus*)	Taiwan	2018	https://news.ltn.com.tw/news/life/breakingnews/2505241 (accessed on 26/07/2022)
4	*Stagmatoptera* sp.	White‐throated Seedeater (*Sporophila albogularis*)	Brazil	2021	https://youtu.be/B6q5uU5oD6k (accessed on 25/07/2022)

Our online searches also found two additional records of praying mantid predation on nestlings. In Taiwan, a Giant Asian Mantid (*Hierodula patellifera*) was recorded preying on a nestling Warbling White‐eye (*Zosterops japonicus*) and in Brazil, an unidentified mantid (*Stagmatoptera* sp.) was recorded feeding on a nestling White‐throated Seedeater (*Sporophila albogularis*; Table 1). Both online observations occurred on trees and nestlings appeared older than those recorded in Iran, i.e., chicks were feathered, however had yet reached the fledgling growth stage. Mantids were also recorded dragging nestlings from the nests.

## 3 DISCUSSION

Our field observations represent the first recorded feeding events between praying mantids and passerine nestlings in nearly 100 years. To date, all mantids recorded feeding on birds have been female (Nyffeler et al., 2017). These unusual observations may be due to two potential explanations. First, during the egg production period, female mantids have been known to use sexual cannibalism as a foraging strategy to increase fecundity, especially those in poor physical condition (Barry et al., 2008). A passerine nestling may provide high nutritional value for a female mantid in poor condition. Therefore, in order to increase fecundity, female mantids may take advantage of nestling birds for nutrient gain. Second, mantids may be attracted to nests with poor sanitation, i.e., lack of removal of fecal sacs by parents, resulting in increased abundances of parasitic or coprophagous insects, e.g., Diptera (Ibáñez‐Álamo et al., 2016). Subsequently, the praying mantids may then prey on nestlings after detecting movements from the young birds.

Despite this, mantids have been known to select optimum hunting sites, e.g., “sitting‐and‐waiting” at vantage points in areas with high prey abundance, observed in Chinese (*Tenodera sinensis*), Bordered (*Stagmomantis limbata*) and European Mantids hunting at hummingbird feeders (Nyffeler et al., 2017). Furthermore, other scents or natural lures may indirectly attract mantids to potential prey sources, e.g., a European Mantid nymph was observed hunting sarcophagid insects that were attracted by the scent of dog feces in Birkirkara, Malta (Cassar, 2020), or in this case to nests with poor sanitation and increased parasitic or coprophagous insect abundances (Ibáñez‐Álamo et al., 2016).

Previous literature and anecdotal evidence have shown that European, Giant Asian and *Stagmatoptera* Mantids are capable of catching and feeding on adult passerines in flight (Browne, 1899; Nyffeler et al., 2017; Ridpath, 1997). However, four bird species (Purple Sunbird, Crested Lark, Warbling White‐eye and White‐throated Seedeater) identified from our observations have not been recorded as prey species of mantids, until now. Hummingbirds (Trochilidae) are preyed upon by mantids in the Americas (Nyffeler et al., 2017), however, are considerably smaller than the species recorded in our observations. For example, an adult Crested Lark weighs approximately 37–55 g, compared with 3–6 g hummingbird species (Kolnegari & Hazrati, 2018; Nyffeler et al., 2017). Nestling passerines more closely resemble the weight of hummingbirds and are at risk of predation by mantids, however, adult passerines are not vulnerable to mantid predation due their increased size. During mist‐netting surveys, a series of observations by Bigas et al. (2006) reported the predation of adult passerines (European Robin *Erithacus rubecula*, Pied Flycatcher *Ficedula hypoleuca*, Cetti's Warbler *Cettia cetti*, Willow Warbler *Phylloscopus trochilus* and European Reed Warbler *Acrocephalus scirpaceus*) by European Mantids in the Ebro Delta, Spain. Despite this, birds were already caught in mist nets and the mantids were not recorded actively catching the adult birds. Therefore, predation of birds by praying mantids may be restricted to nestlings and only adults of species which are exceptionally lightweight, such as hummingbirds.

The European Mantid is considered an invasive species across parts of its current distribution (Jones & DiRienzo, 2018) and continues to expand across Europe, Asia, Africa and North America (Anderson, 2019; Battiston & Fontana, 2010; Lin & Griebeler, 2015). The species has been shown to be particularly good at occupying new habitats (Prete et al., 1999; Walther et al., 2009; Zieliński et al., 2018) and depredation on nestling birds may become a future conservation issue only if nestlings of species that occur in extremely low population sizes are preyed upon. At present, there are no data to suggest this, however, more research is needed on the impacts of predatory insects on threated vertebrate populations.

Observations reported here represent the first recorded feeding events between praying mantids and passerine nestlings in nearly 100 years. Ecological explanations for these events remain speculative and further field surveys and research is required in order to further increase our knowledge and understanding on predator–prey relationships between trophic groups.

## AUTHOR CONTRIBUTIONS

**Mahmood Kolnegari:** Conceptualization (lead); data curation (lead); investigation (equal); methodology (lead); visualization (lead); writing – original draft (lead); writing – review and editing (equal). **Antonio Fasano:** Investigation (equal); validation (equal); writing – review and editing (supporting). **Khalil Zareie:** Data curation (supporting); investigation (supporting). **Connor T. Panter:** Investigation (equal); writing – review and editing (lead).

## FUNDING INFORMATION

No funding was required for this study.

## Data Availability

All observation data presented in this work are included in Table 1.
